# Aphid infestation in the phyllosphere affects primary metabolic profiles in the arbuscular mycorrhizal hyphosphere

**DOI:** 10.1038/s41598-018-32670-1

**Published:** 2018-09-27

**Authors:** Carmina Cabral, Bernd Wollenweber, Carla António, Ana Margarida Rodrigues, Sabine Ravnskov

**Affiliations:** 1 Aarhus University, Department of Agroecology, Forsøgsvej 1, DK-4200 Slagelse, Denmark; 2 Plant Metabolomics Laboratory, Instituto de Tecnologia Química e Biológica António Xavier-Universidade NOVA de Lisboa (ITQB NOVA), Avenida da República, 2780-157 Oeiras, Portugal

## Abstract

While effects of (a)biotic stress events in the phyllosphere have been studied intensively, possible influences of stress on the arbuscular mycorrhizal hyphosphere has scarcely been investigated. We hypothesised that stress challenge in the phyllosphere could alter primary metabolite profiles of the hyphosphere - the mycelial network connecting plants. Donor plants, connected to receiver plants by mycelial networks, were aphid-challenged during 84 h. Primary metabolite profiles in the hyphosphere were investigated. Gene-expression of plant defence gene PR1 was measured in one of the receiver plants during the challenge. Hexose levels in the hyphosphere increased when donor plants were aphid-challenged. This change in metabolic profile was influenced by leaf sampling from receiver plant. PR1 expression increased in donor plants 48 h after challenge, and consequently 60 h after, in receiver plants. We conclude that aphid infestation of donor plants modified primary carbon metabolism in the hyphosphere. Plant defence response in receiver plants, occurred 12 h after detection of response in the aphid-challenged donor plants. While this work is the first to reveal primary metabolic profiles of the AM hyphosphere, more work is needed to elucidate the possible role of transient changes of hexose metabolism in stress response and signalling processes in the hyphosphere of connected plants.

## Introduction

Arbuscular mycorrhizal fungi (AMF) are globally distributed biotrophic organisms, depending on living root tissue for carbohydrate supply to complete their lifecycle^1,2^. AMF form mutualistic symbiotic relationships with approximately 80% of vascular plants, which they supply with mineral nutrients from the soil^3^. The effectiveness of this bidirectional nutrient transfer has been reported to be dependent *inter alia* on the functional compatibility between AMF -and host plant genotypes^4–6^. The AM symbiosis can influence the nutritional status of the host plant^6–9^, may improve disease-tolerance^10^ and alleviate abiotic stress in plants^11–14^.

In natural environments, low host preference specificity and the ability to anastomose assures that individual fungi form extensive and functional AM mycelial networks, ranging up to 10 m in diameter^15^ and connecting different plant species^16,17^, thereby building interconnected plant communities^18^. Through these AM underground networks, plants can interact with each other by exchanging nutrients, thereby influencing plant growth^19,20^. Several reports have focused on the possibility of unequal nutrient transfer inside these networks, which would increase competition between plants^21,22^ by shifting carbon into roots of neighbouring plants *via* mycelial networks, where the shift may be skewed towards one of the involved plant species^23^. This shift has been postulated to be dependent on the functional compatibility of the AM genotype and its host plants^24^.

Moreover, mycelial networks have been proposed to function as underground communication “channels”^25^. Underground signals have been proposed to be able to travel through the AM mycelial network, there is evidence that after a challenge to “donor” plants, defence responses, both to fungal pathogens as well as to herbivory, are induced in “receiver” plants connected to the donor by AM mycelial networks^26–28^. Interestingly, induction of defence responses in “receiver” plants seem dependent on the type of defence response induced in the “donors”^28^. In a comparable study, aphid infestation in “donor” plants (*Vicia faba* L.) was reported to induce the production of volatile organic compounds (VOC) in “receiver” plants connected through AM mycelial networks^26^. However, although VOC production and changes in gene expression in plants have been reported^26–28^, the possible role of the AM fungal network connecting the plants in signalling of the stress response remains elusive, and the reported alterations have only been evaluated at the plant level - the phyllosphere.

There are two major types of plant immune response: a response against pathogen-associated molecular patterns (PAMPS) - PAMP-triggered immunity (PTI)^29^, and an effector-triggered response (ETI) where the host is able to sense effector molecules and in which specific antimicrobial resistant proteins (R proteins), are secreted towards the specific pathogen or pest infection, in order to quell PTI^29^. This triggering of PTI defence responses is related to a wide array of different plant processes, which can involve induction of physical changes and biochemical responses, such as the production of salicylic acid (SA) or jasmonic acid (JA)^30^. Triggering this wide array of defence responses at the same instance necessitates also the re-allocation of large amounts of energy- and reduction equivalents (ATP and NADH), which are normally allocated to plant growth and to general metabolism^31^. This reallocation of energy to plant defence responses has been reported to be related to changes in the primary metabolism in the phyllosphere^32^. But interestingly, not in the hyphosphere, consisting of the mycelium connecting the plants, as well as the associated microbial community.

Here, we hypothesise that if a challenge in the phyllosphere of a “donor” plant elicits a response in the phyllosphere of AMF connected “receiver” plants, then the hyphosphere should reflect this challenge by a modification of its primary metabolic profile. Specifically, the objectives of this study were to use a purpose-build experimental system to assess: (i) whether the primary metabolic profile of the hyphosphere is changed after aphid challenge of the donor plant; (ii) if an aphid challenge to the phyllosphere of “donor” plants elicits an induction of salicylic acid (SA) - dependent defence pathways in “receiver” plants; (iii) if abiotic stress of the phyllosphere combined with aphid challenge influences the metabolic profile of the hyphosphere.

## Results

### Plant growth, AMF colonization, microbial biomass in root-free compartments and aphid reproduction on donor plants

There was no significant effect of AMF colonization on dry weights and heights of donor and receiver plants (Table 1). Root length was significantly lower in M+ receiver plants, while no significant differences were found in donor plants (ANOVA, *P*-value = 3.07e-07, Table 1). All *Aphis fabae* individuals reproduced during the challenge, with no significant difference in the reproduction rates between M+ and M− donor plants (Table 1). There was no effect of AMF colonization or aphid infestation in light-use efficiency parameters of the receiver plants (Supplementary Figs S1 and S2, available online).

**Table 1:** Growth parameters of *Vicia faba* L. and *Aphis fabae* Scop. reproduction rates. Donor plants columns represent plants from the donor compartment inoculated (M+) or not (M−) with AMF.

	Donor plants		Receiver plants	
	M−	M +	M−	M+
Dry weight (g)	3,19a^a^	3,82a	2,61a	3,19a
Height (cm)	65,16a	73a	65,35a	66,43a
Root dry weight (g)	0,91a	0,99a	1,14a	1,25a
Total root length (m)	747,93a	622,58a	652,78a	352,78b
Aphid reproduction (n)	28,6a	29,4a		

Receiver plants columns represent plants from the receiver compartments, in which AM networks via the donor plant were (M+) or not (M−) established. Values are means (Donor plants: n = 5; Receiver plants: n = 10). ^a^In each row, followed by the same letter are not significantly different by Tukey’s HSD with a *P*-value fdr (false discovery rate) correction.

**Figure 1:**
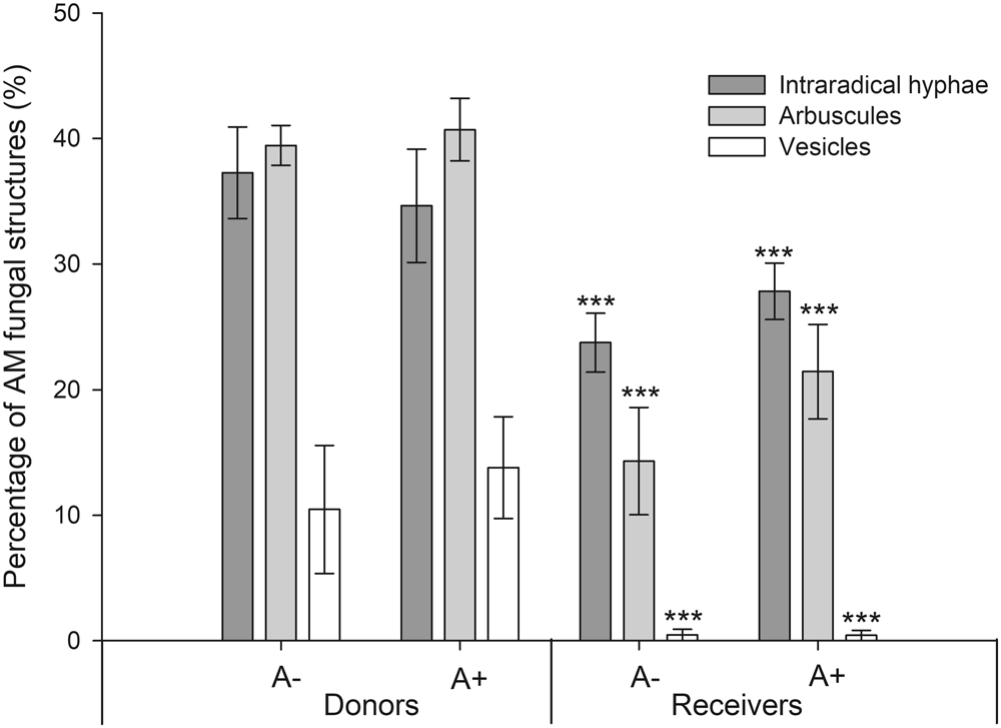
Percentage of fungal structures in donor plants and in receiver plants. These are categorized into: intraradical hyphae, arbuscules and vesicles. Donor plants in these systems were (A+) infested with aphids or not (A−). Comparison between donor and receiver plants was tested and statistical significance is represented by *** (ANOVA, p-value < 0.001, Donor plants: n = 5; Receiver plants: n = 10) Error bars represent +− SE of the mean.

No AMF structures were found in M− donor plants, and no structures were found in M− receiver plants either, confirming that no AM network was established through the root-free compartments in the M− treatment. The percentage of intraradical hyphae, arbuscules and vesicles was significantly higher in donor plants, when compared to receiver plants (Fig. 1). There was no significant effect of aphid infestation of donor plants in the percentage of AM fungal structures in donor or receiver plants (Fig. 1).

AM fungal biomass in the root-free compartments was determined by the abundance of the biomarker fatty acid 16:1w5c. Background content of fatty acid 16:1ω5c in the M− root-free compartments was subtracted from values measured in growth substrate from root-free compartment in M+ treatments. Phospholipidic-derived fatty-acid (PLFA) values were 0.90 ± 0.19 nmol 16:1w5 g^−1^ growth substrate, whilst neutral lipid fatty-acids (NLFA) values were 2.18 ± 1.04 nmol 16:1w5 g^−1^ growth substrate. There was no significant difference in biomass of AMF in root-free compartments of A− and A+ treatments. Signature PLFAs: 15:0iso, 15:0anteiso, 16:0iso, 17:0iso and 17:0anteiso, relative to signature internal standard PLFA 19:0 were used to determine the abundance of gram positive bacteria. Gram positive bacteria abundance, was significantly higher in M+ root-free compartments, 0.062 ± 0.007, than in M− root-free compartments, 0.046 ± 0.007 (ANOVA, *P*-value = 0.007). There were no significant differences in the abundance of gram positive bacteria between A− and A+ treatments. The abundance of gram negative bacteria, actinomycetes and protozoans was below detection limit in the root-free compartments.

### Relative gene-expression of PR1

**Figure 2:**
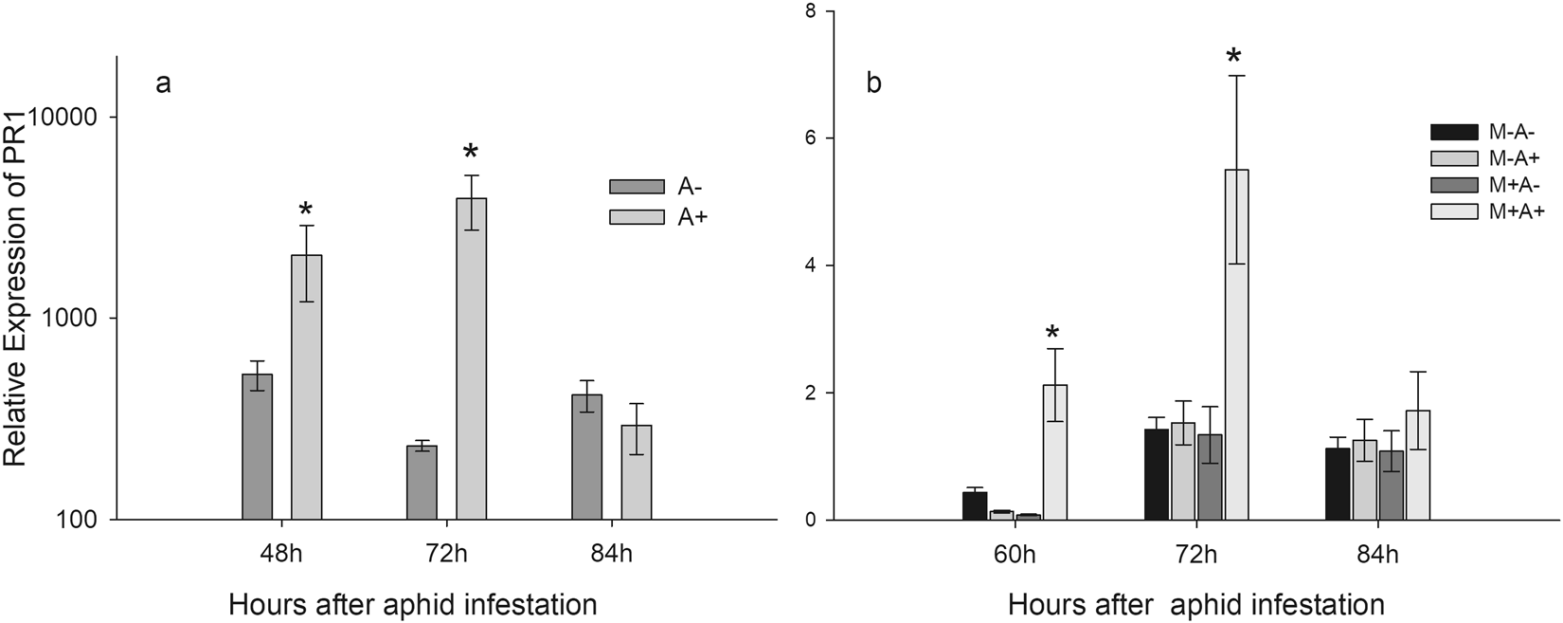
(**a**) Relative expression of PR1 in donor plants 48, 72 and 84 hours after aphid infestation. Plants were infested with aphids (A+) or not (A−). (**b**) Relative expression of PR1 in receiver plants 60, 72 and 84 hours after aphid infestation of donor plants. AM networks via the donor plant were (M+) established in receiver plants or not (M−). Donor plants in these systems were (A+) infested with aphids or not (A−). Statistical significance between treatments inside timepoints indicated by * (ANOVA, *P*-value < 0.005, n = 5). Error bars represent +− SE of the mean.

The relative expression of PR1 in the SA- dependent defence pathway was measured in donor and receiver plants during the course of the aphid challenge, and relative expression was determined in relation to ELF1-α. In donor plants challenged with aphids (A+), relative expression of PR1 increased 2047 fold and 3926 fold at 48 and 72 hours after aphid infestation, respectively (Fig. 2a). In receiver plants, relative expression of PR1 increased 2.12 and 4.4 fold in M+A+ plants at 60 and 72 hours, when compared to relative expression in receiver plants of the remaining treatments (Fig. 2b).

### Primary metabolism profiles in root-free compartments

**Figure 3:**
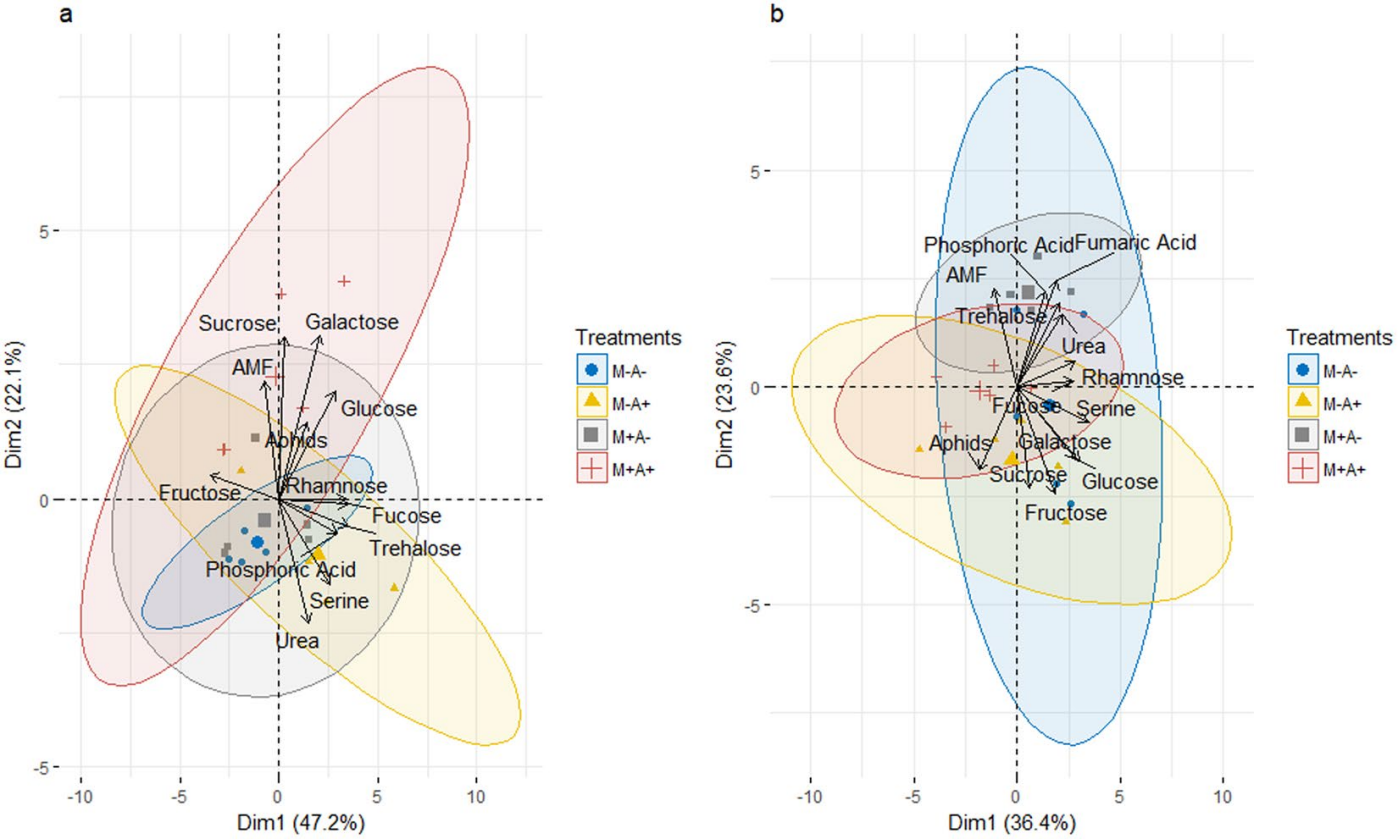
(**a**) PCA Biplot of relative primary metabolite levels normalised to ribitol as an internal standard and the dry weight of the samples, in undisturbed root-free compartments. Root-free compartments had established AM networks (M+) or not (M−). Donor plants in these systems were (A+) infested with aphids or not (A−). (**b**) PCA Biplot of relative primary metabolite levels normalised to ribitol as an internal standard and the dry weight of the samples, in disturbed root-free compartments. Root-free compartments had established AM networks (M+) or not (M−). Donor plants in these systems were (A+) infested with aphids or not (A−). Ellipses represent 95% confidence interval of scores distribution per treatment, n = 5.

In total 10 putative primary metabolite signatures were found to be present in root-free compartments: galactose, sucrose, fructose, glucose, urea, fucose, trehalose, rhamnose, serine and phosphoric acid (Table 2, Supplementary Table S1). Primary metabolic profiles in growth substrate of the two root-free compartments in each growth system were analysed independently. In order to highlight the ecological significance of aphid infestation, the response of primary metabolites to aphid infestation of donor plants in M+A+ root-free compartments was presented as fold-changes related to M+A− root-free compartments (Tables 2 and 3), whereas a full dataset in presented online in Supplementary Tables S1 and S2. There was a statistically significant effect of AMF colonisation (ANOVA, *P-*value = 0.014), aphid infestation of the donor plants (ANOVA, *P*-value = 0.0052) and an AMF*aphids interaction (ANOVA, *P-*value = 0.0022) on the levels of galactose (Table 2, Supplementary Table S1). Post-hoc analysis showed that levels of galactose and sucrose were higher in M+A+ compartments, while phosphoric acid levels were lower in compartments colonised by AMF (Table 2, Supplementary Table S1). In the PCA biplot of primary metabolites in undisturbed compartments, dimensions 1 and 2 accounted for 47.2% and 22.1% of the variation, respectively (Fig. 3a). AMF mycelium in root-free compartments or challenge of donor plants with aphids as single factors did not have an effect on overall primary metabolic profiles of the undisturbed root-free chambers (Fig. 3a). But, in combined treatments with both AMF mycelium and aphid challenge of donor plants (M+A+) there was a separation in metabolite profiles, as exemplified by the loadings of galactose (ANOVA, *P*-value = 0.002), and further indicated by the loadings of sucrose (ANOVA, *P*-value = 0.065).

**Table 2:** Fold changes of relative primary metabolite levels in M+A+ relative to M+A− treatment, normalised to the internal standard (ribitol) and dry weight of the samples, in undisturbed root-free compartments. AM networks via the donor plant were (M+) established in receiver plants or not (M−).

Classes	Metabolites	M+A−	M+A+
Amino acid	Serine	1 ± 0.23	0.45 ± 0.45
Sugars	Fructose	1 ± 0.10	1.74 ± 0.60
	Fucose	1 ± 0.23	0.99 ± 0.16
	Galactose	1 ± 0.18	3.00 ± 0.24
	Glucose	1 ± 0.19	1.15 ± 0.54
	Rhamnose	1 ± 0.50	1.00 ± 0.18
	Sucrose	1 ± 0.67	4.50 ± 1.70
	Trehalose	1 ± 0.16	0.99 ± 0.27
Others	Phosphoric Acid	1 ± 0.26	0.73 ± 0.22
	Urea	1 ± 0.13	0.88 ± 0.04

Donor plants in these systems were (A+) infested with aphids or not (A−). Values are means ± standard error (SE). n = 5.

**Table 3:** Fold changes of relative primary metabolite levels in M+A+ relative to M+A− treatment, normalised to the internal standard (ribitol) and dry weight of the samples, in disturbed root-free compartments.

Classes	Metabolites	M+A−	M+A+
Amino acid	Serine	1 ± 0.11	0.67 ± 0.10
Sugars	Fructose	1 ± 1.00	0.96 ± 0.06
	Fucose	1 ± 0.10	0.74 ± 0.10
	Galactose	1 ± 0.07	1.04 ± 0.20
	Glucose	1 ± 0.08	0.85 ± 0.13
	Rhamnose	1 ± 0.10	0.66 ± 0.09
	Sucrose	1 ± 0.14	1.6 ± 0.67
	Trehalose	1 ± 0.15	0.66 ± 0.10
Others	Fumaric Acid	1 ± 0.43	0.26 ± 0.06
	Phosphoric Acid	1 ± 0.23	0.44 ± 0.06
	Urea	1 ± 0.27	0.55 ± 0.12

AM networks via the donor plant were (M+) established in receiver plants or not (M−). Donor plants in these systems were (A+) infested with aphids or not (A−). Values are means ± standard error (SE). n = 5.

Leaf sampling of the receiver plants shifted the primary metabolic profiles in disturbed root-free compartments, 11 putative primary metabolite signatures were found in these compartments: galactose, sucrose, fructose, glucose, urea, fucose, trehalose, rhamnose, serine, phosphoric acid and fumaric acid (Table 3). In the PCA biplot for primary metabolites, dimensions 1 and 2 accounted for 36.4% and 23.6% of variation, respectively, with the loadings including fumaric acid, which was not detected in undisturbed root-free compartments (Fig. 3b). There was an overlap between treatments in the primary metabolic profiles of the disturbed root-free compartments (Fig. 3b). However, there was a punctual statistically significant effect of aphid infestation of the donor plants (*P*-value = 0.01), that seemed to lower the levels of phosphoric acid, as further evidenced in the post-hoc analysis (Supplementary Table S2). There was no statistical significance in the levels of galactose in the disturbed compartments (Fig. 3b).

## Discussion

In the present study, modifications of primary metabolite profiles occurring in the hyphosphere of AMF interconnected plants are reported. Interestingly, from the metabolic signatures identified in the hyphosphere, carbohydrates were the only metabolites found to increase in response to an aphid infestation of the phyllosphere, as opposed to metabolites known to be involved in nutrient uptake and transport, such as glutamate and arginine as N transport forms in the extraradical mycelium (ERM)^9,33^. However, impact of AMF colonisation in plant roots has been reported at plant primary metabolism level, namely in the amino-acid and carbohydrate metabolism^34^, therefore, the observed changes in the carbohydrate pools in the ERM might be related to an increased carbohydrate metabolism in the roots intraradical mycelium (IRM).

It has been shown that in AMF, hexoses from the host plant are transferred to the IRM, where they are converted into storage lipids, which in turn are translocated into the ERM. In the ERM, these storage lipids are converted into hexoses *via* catabolism of triacylglicerides (TAGs) and the glyoxylate cycle^35^. Importantly, the ERM cannot absorb exogenous hexoses, and it is, therefore dependent on this storage lipid translocation, to assure fungal growth and proliferation of the ERM^36^. In this study, the increase of these ERM hexose pools in the hyphosphere connecting aphid infested plants could indicate that the translocation of AMF storage lipids as well as glycogen from the plants IRM to the common ERM was higher after aphid infestation, which might be related to the induction of the PTI defence response in the donor plant^31^. Furthermore, direct lipid allocation from the plant to the AMF has recently been reported^37^, which may provide an alternative signalling mechanism between plant and AMF.

The plant defence response to aphid infestation has been shown to be dependent on the SA-pathway^38^, meaning that a PTI defence response would affect primary metabolism via reallocation of resources and energy from plant growth to defence^31,32^. It is therefore possible that an increased resource-demand for PTI in response to aphid infestation in AM donor plants has led to an increase in the translocation of storage lipids from the IRM to the interconnected ERM^35^, correlating with the increase of the hexose pool in the hyphosphere. On the other hand, as the energy and resource demands of the PTI response are high^31^, the infested plants might stop or reduce the available hexose pool for transfer to the fungus. In this way, the IRM metabolism might be disrupted, increasing the synthesis of storage lipids, translocating them towards the ERM and increasing the hexose pool. By increasing the synthesis of storage lipids, the fungus might safeguard itself in regards to the death of a diseased host, and this may account for the changes in primary metabolic profile in the hyphosphere of interconnected aphid infested plants. AMF have, previously, been reported to induce growth depressions, related to several causes, namely increasing the lipid retention in the IRM, by increasing the number of vesicles^39,40^. The results presented in this study seem to support these hypotheses, however, further studies using ^13^C & ^14^C labelling and metabolic flux analysis would be necessary to investigate them in detail.

AM symbiosis has been reported to have an impact on plant defence responses against insect pests, either indirectly, by influencing plant nutrition, or directly, by modulating gene expression, or by stimulating the plant immune system - mycorrhiza-induced resistance^41,42^. In the current study, no physiological or agronomical differences were detected between aphid infested and non-aphid infested AM plants. However, mycorrhiza-induced resistance has also been reported to be related to the mycorrhiza-associated bacteria in the hyphosphere^41^. This could have played a part in the increased hexose pool in the hyphosphere of interconnected aphid infested plants. Nevertheless, if the mycorrhiza-associated bacteria had significant influence on the hexose pools in the current study, a higher bacterial biomass, due to their fast replication rate, would be expected in the hyphosphere of interconnected aphid infested plants. But, as no significant differences in biomass of bacteria were detected in response to aphid infestation, this would have to be investigated in future detailed studies involving ^13^C & ^14^C labelling.

Furthermore, leaf sampling on the receiver plants caused a shift in the hyphosphere primary metabolite profiles. This might be due to the induction several stresses at the same time (abiotic and biotic), causing cross-talk of several defence pathways^43^. The occurrence of signalling related to abiotic stress has been shown in ectomycorrhizal mycelial networks^44^, but to date no evidence of this has been found in AM mycelial networks. In the current study, induction of abiotic stress, caused by leaf sampling, together with the biotic stress from aphid infestation, might have caused several other processes to take place in the soil-fungus interface, modifying the metabolism of the hyphosphere. This theory is supported by the demonstration that topping, which is a common practice in tobacco cultivation, can cause changes in plant primary metabolism^45^. As such, these metabolic shifts might be transmitted to the hyphosphere, possibly obscuring the changes seen in undisturbed compartments, with only one stress type.

In the present study, we show that an increase in the PR1 relative expression levels in the infested donor plants at 48 h is followed by a consequent increase in the interconnected receiver plants at 60 h, indicating that there is signalling from the donor to the receiver plant, causing the induction of defence in the receivers. These results correlate with previous studies^27,28^, where the expression of several defence genes was shown to increase in receiver plants connected to challenged donor plants through AM networks. In the study by Song *et al*.^27^, there is an increase in the relative expression of PR1 in receiver plants starting 65 h after pathogen inoculation of the donor plants, and accumulating up to 140 h. This differs from the results presented in the current study, where PR1 relative expression peaks at 72 h, and declines at 84 h. Although PR1 relative expression starts increasing in receiver plants at 60 h post aphid infestation, it peaks at 72 h, followed by a decline in the response over time, which is consistent with the model presented in the meta-analysis performed by Johnson and Gilbert^46^, where the timings of receiver plant responses were synthesised over different systems and studies.

Thus, it is clear that the timing of the signalling through the hyphosphere, as demonstrated by the increase in the relative expression of defence- gene levels in plants, differed between studies, host plants and biotic stress factors. Different AMF genotypes have been reported to differentially influence carbon metabolism in roots of the same host plant^47^. It might, therefore, be pertinent to further investigate if an impact of the host plant-AMF genotype combination is related to the timing and effectiveness of the signalling between interconnected plants. Namely, in regards to the speed of cytoplasmic streaming of solutes through the AM hyphae^48^.

The reported changes in relative expression of PR1 in both donor and receiver interconnected plants indicate that a signalling event has taken place through the hyphosphere. Accordingly, it is possible to hypothesise that the changes in the primary metabolic profile of the hyphosphere of interconnected plants might be related to the modulation of the expression of PR1 on the receiver plants, after signalling from the donor plant. Carbohydrates have previously been reported to act not only as nutrients, indispensable for growth and plant health, but also as signals, which modulate gene-expression^31,49^. This might indicate that the observed changes in the hexose pools of the hyphosphere could have been related to the induction of plant defence genes (PR1) in the connected plants. However, as there is no transfer of carbon from the AM fungus to the root^50^, it is unlikely that sugars in the hyphosphere of interconnected plants might have been involved in the modulation of gene-expression inside the plants. Therefore, the nature of the signalling molecule traveling the AM mycelial networks remains elusive.

A recent interesting insight and perspectives paper^51^ has raised interesting points of possible consequences of mycelial networks for the AM fungus itself, such as securing carbon pools from selected donors. In addition, signal transfer from a donor plant to an interconnected second plant could prepare or ‘prime’ that plant against a potential attack. However, to date, no data is available on these propositions, and future efforts could elucidate these ecological interactions.

In conclusion, the present study shows that a stress event in the phyllosphere was followed by changes in the primary metabolite profiles in the hyphosphere of interconnected plants, which may be related to the energetic needs of the PTI defence response. Furthermore, as these changes were not detected in hyphosphere compartments connecting plants, where leaf sampling had taken place, this study indicates that a possible cross-talk of defence pathways may influence primary metabolite profiles of the hyphosphere. Adding to previous studies, evidence of signalling through mycelial networks has also been shown, through plant defence gene expression in the phyllosphere of interconnected plants. However, the nature of the possible signalling components travelling through these networks remains elusive.

The results presented demonstrate the need for further studies in this field, as the changes in primary metabolism of the hyphosphere in response to a pest attack on the phyllosphere open up several new questions such as: are the hexoses being translocated from the IRM of infested plants to the ERM of connected plants? Will the infested plant cease/reduce carbon-transfer to the AMF? Are hexoses being translocated from the IRM of receiver plants to the hyphosphere to sustain fungal growth? Further studies using ^13^C labelling and ^13^C metabolic flux analysis may shed a light on these questions. In addition, the changes in relative expression of PR1, in both infested and receiver plants support evidence that signalling between plants occurs through the AM mycelial network. Further studies, for example with focus on changes to the secondary metabolism in the hyphosphere, might be able to unveil the nature of the signalling molecules able of travelling through the underground networks.

## Material and Methods

### Experimental setup and growth conditions

A greenhouse experiment with the *Vicia faba* L. (Faba bean) cultivar “Boxer”, selected due to its uniform growth, was performed at Aarhus University, Denmark (55°19′N, 11°24′E) from January to March 2017. The experiment followed a full factorial design with two factors: inoculation of donor plants with AMF (M+) or not (M−), and infestation of donor plants with aphids (A+) or not (A−). Each combination of factors consisted of five biological replicates.

**Figure 4:**
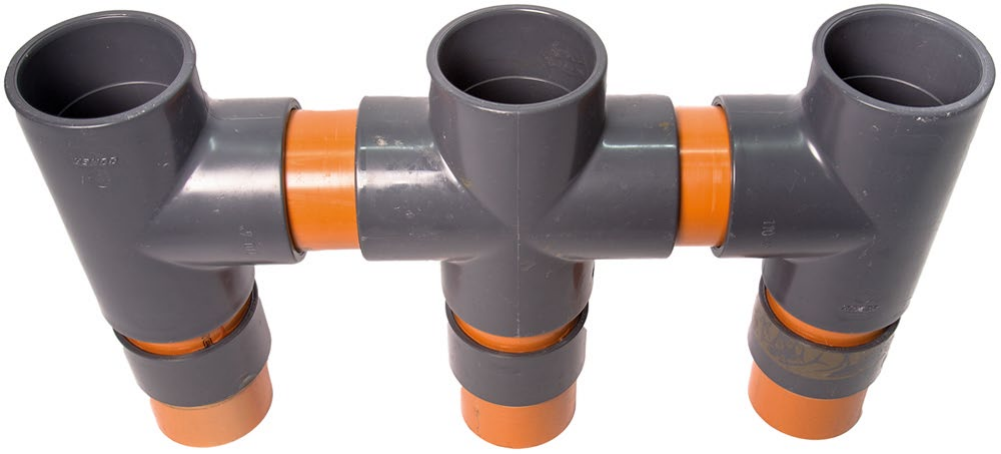
Plant growth system. 3.4 L plant pots (110 mm diameter) are connected through a 1.2 L (110 mm diameter) root-free chamber. Donor plant pot is at the center and connected to one receiver plant pot at each side. Photograph taken by Charlotte Knudsen.

Modified cross-tube plant growth systems, adapted from^5^ were used. A 3.4 L donor plant pot was placed at the centre and connected to two 3.4 L receiver plant pots on either side, by 1.2 L root-free chambers (Fig. 4). The two root-free compartments in each growth system were either “undisturbed” or “disturbed”. The disturbed root-free compartment connected donor-plant pots with receiver-plant pots, where plants were used for leaf sampling to gene expression analyses, whereas the other receiver plant in same growth system was left undisturbed.

A total of 20 plant growth systems were filled with a 1:2 mixture of soil: sand twice disinfected at 80 °C/48 H. Initial plant available nutrient concentrations and final growth substrate nutrient concentrations were as described in detail in^11^. In the donor plant compartments, from a total of 5 kg growth substrate, the upper 3 kg were either mixed with 6 g (2400 spores) of *Rhizophagus irregularis* (Błaszk., Wubet, Renker & Buscot) C. Walker & A. Schüßler^52^ lyophilized inoculum (Symbiom^©^) or with the diatomite carrier used for the AMF inoculum (M+ and M− treatments), whilst receiver plant compartments were filled with 4.5 Kg growth substrate. The root-free compartments were filled with 800 g of 1:3 disinfected soil:sand mixture, as follows: 150 g followed by a nylon mesh, 500 g followed by another nylon mesh, and additional 150 g, with the purpose of creating 1 cm buffer zones to plant compartments at each end of the root-free compartment connecting donor and receiver plants. Growth substrate from the buffer zones was discarded at harvest time. After filling, each end of the root-free compartment was closed with a 20 µm nylon mesh and inserted into the plant compartments, creating a 6 cm distance between donor and receiver plant compartments.

Growth systems were watered to 100% soil relative water content, as previously calculated for the same soil type^11^ and left for a week to allow for AMF inoculum incubation prior to germinant seed insertion. Seeds were scarified and sterilized in lab conditions with a sulphuric acid (92%) and sodium hypochlorite (8%) treatment, posteriorly incubated at 24 °C for 2 days before sowing. Plants were grown, for 9 weeks at 16:8 light/dark, and day:night temperature of 24:20 °C.

### Aphis fabae population and aphid challenge of donor plants

*Aphis fabae* Scopoli were collected from a Danish faba bean (*Vicia faba* L.) field and the population were maintained during the course of the experiments, by providing the aphids with fresh *Vicia faba* L. plants once per week, assuring survival and reproduction. Donor plants were challenged with aphids eight weeks after sowing and were exposed to the aphids for 84 h. For the challenge, 30 adult *Aphis fabae* Scop. individuals were selected and placed inside custom-made clip-cages^53^ on the A+ donor plants, on the 3^rd^, 4^th^, 6^th^, 7^th^ and 8^th^ leaves, while empty clip-cages were used for the A− donor plants. Immediately after mounting of clip cages, donor plants were covered with sealed plastic bags to avoid possible signalling effects by volatile organic compounds over the air to the receiver plants. During the aphid challenge, leaves were collected from one of the two receiver plants every 12 h and from extra donor plants (set apart from the main experiment) every 24 h, flash-frozen in liquid nitrogen, freeze-dried, ground into a homogenised fine powder for subsequent analysis. At harvest time, the clip-cages and leaves were recovered and the final number of aphid individuals was counted in order to determine their reproduction rates.

### Measurement of Light-use efficiency parameters

During the three days of aphid challenge applied at eight weeks after sowing, light-use efficiency parameters were measured on the 9^th^ leaf of the “receiver” plants after 30 min of dark adaptation, using a pulse-modulated fluorometer (Mini-pam, Walz, Elfeltrich, Germany). Light-use efficiency parameters were posteriorly determined as Photosystem II yield (ɸPSII), Electron transfer rate (ETR) and non-photochemical quenching (NPQ).

### Gene-expression assays in faba bean plants

Differential expression of selected plant genes was verified by RT-qPCR using the RNA samples isolated from leaves of one of the receiver plants in each growth system. Leaves were sampled from the same plants at 12 hour intervals after aphid infestation The *elongation-factor 1-α* Elf1-α gene was used as a house-keeping gene, for normalization of expression, using the 2^−ΔCT^ method^54,55^. Total RNA was extracted from faba bean leaves, using an RNA NucleoMag Kit (744350.1 MACHEREY-NAGEL GmbH & Co. KG), according to manufacturer’s instructions. First strand cDNA was synthesized from 1 mg of total RNA using a High activity cDNA Reverse Transcription Kit (4368814 Applied Biosystems®) according to the manufacturer’s instructions. The primers for target genes *pathogenesis-related protein* (PR1) (*VfPR1-forward*: CAGTGGTGACATAACAGGAGCAG; *VfPR1-reverse*: CATCCAACCCGAACCGAAT), and Elf1-α (*VfELF1A-forward*: GTGAAGCCCGGTATGCTTGT; *VfELF1A-reverse*: CTTGAGATCCTTGAC TGCAACATT), were selected from the literature^56,57^.

The RT-qPCR reactions were carried out using 1 µL (10 mM) of each specific primer pair, 2 µL cDNA, 6 L of the SYBR green master mix (Quanti Tech SYBR Green kit, Qiagen, Gmbh Hilden, Germany) diluted to a final volume of 12 µL with RNase-free water. In the negative control cDNA was replaced by RNase free water. The reactions were performed on a Applied Biosystems® ViiA™ 7 Real-Time PCR System. The programme used for RT-qPCR was as follows: heating step of 2 min at 50 °C, 10 min initial denaturation at 95 °C, followed by 40 cycles of denaturation for 15 s at 95 °C, annealing for 1 min at 60 °C. The fluorescence signal was measured immediately after incubation for 1 min at 60 °C following the annealing step. At the end of the cycles, melting temperatures of the PCR products were determined between 60 °C and 95 °C. The specificity of amplicons was verified by melting curve analysis. No less than 3 biological replicates (from different plants) were independently carried out by treatment and time-point, and two technical replicates (2 reactions with template from same plant) of each were considered for relative expression.

### Harvest, plant fresh- and dry weights

Plants were harvested 84 h after aphid infestation. Root-free compartments were rotated and dislodged from the plant pots, the buffer zones were discarded, and the remaining growth mixture from the root-free compartments was immediately submerged into liquid nitrogen, to stop all metabolic processes and prevent metabolite degradation. Afterwards, plants were divided into above- and belowground plant parts. Plant height was measured, and all plant material was weighed, frozen, freeze-dried, weighed and stored at −20 °C, prior to further chemical analysis.

### Root staining, root length and AMF colonization analysis

All root material was carefully rinsed with tap water and cut into 1 cm pieces. Samples of 0.8 g fresh weight were taken for determination of AMF colonization. These samples were cleared in 10% KOH and stained in 5% inkblue acetic acid, adapted from^58^ and stored in glycerol. AM fungal colonization was assessed by microscopy at 16x magnification, using the point-intersect method^59^, and AM fungal structures were classified as: intraradical hyphae, arbuscules and vesicles. The remaining root material was frozen, freeze-dried and stored at −20 °C, prior to further analysis.

### AMF biomass in root-free compartments

Four grams of freeze dried growth substrate from root-free compartments was used for quantification of the AMF by the fatty-acid signature 16:1ω5. Samples were submitted to a methanol/chloroform lipid extraction protocol according to^60^, followed by fractionation in silica columns, with collection of the polar and neutral phases. Both phases were evaporated under nitrogen flow, and 25 µg ml^−1^ nonadecanoic acid methyl ester (19:0) were added to each sample as an internal standard. Both lipid phases were subjected to mild alkaline methanolysis^61^, to achieve transformation into free fatty acid methyl esters. Analysis was performed using a HP Chemstation and a HP5890 CG fitted with a 25 m fused in silica capillary column (HP part 19091B-102), using hydrogen as a carrier gas, injector temperature was 250 °C and detector temperature was 300 °C. The temperature program was set as: 170 °C as initial temperature, increasing up to 270 °C at 5 °C min^−1^, 1 µL of sample preparation was injected into the Chemstation. The analysis was performed with the software package Sherlock Version 6.0 (MIDI Inc).

### Extraction and quantification of primary metabolites from root-free compartments

Primary metabolites were extracted using a methanol/chloroform extraction protocol according to^62^. A total of 4 g dry weight (DW) of finely homogenized growth substrate from root-free compartments was weighed into 15 mL falcon tube and diluted in 2800 µL ice-cold 100% (v/v) methanol with 120 μL of ribitol (0.2 mg mL^−1^ ribitol in water) as an internal standard. The mixture was vortex-mixed, incubated for 15 min on an orbital shaker (Brunswick™ Innova® 44) at 250 rpm for 15 min at 70 °C. Subsequently, each tube was centrifuged at room temperature at 14,000 *g* for 10 min. The supernatant was transferred to a new 2.0 mL safe-lock Eppendorf tube, mixed with 750 μL chloroform and 1500 μL water, and vortex mixed. Samples were centrifuged at 4000 rpm for 15 min at room temperature. A total of 1000 μL of the polar (upper) aqueous/methanol phase were evaporated to dryness using a centrifugal concentrator (Vacufuge Plus, Eppendorf) and stored at −80 °C. Primary metabolites were derivatised and analysed using an established GC-TOF-MS protocol^62^ at the Biochemistry and Plant Biotechnology Laboratory (University of Malaga, Spain). GC-TOF-MS chromatograms were subsequently evaluated at the Plant Metabolomics Laboratory (ITQB NOVA, Oeiras, Portugal), using TagFinder^63^. Analytes were manually identified using the TargetFinder plug-in of the TagFinder software and a reference library of ambient mass spectra and retention indices from the Golm Metabolome Database (http://gmd.mpimp-golm.mpg.de/)^64,65^. GC-TOF-MS relative primary metabolite levels were normalized to the internal standard (ribitol) and calculated according to specific dry weight of the samples.

### Statistical analysis

The statistical analysis was performed with R-software^66^. Initially, the data was tested for normality and homoscedasticity, using Shapiro-Wilks and Bartlett’s test, respectively. Values expressed in percentage, relative values as well as ratios were log-transformed, before statistical analysis, in order to fit the normality and homoscedasticity assumptions of the analysis of variance (ANOVA). Two-way ANOVA was used to assess differences between treatments, and post-hoc analysis for mean comparison at a 95% confidence level was performed in terms of Tukey’s HSD, with a false discovery rate correction on the *P*-values. Principle Component Analysis (PCA) on the relative primary metabolite levels was performed using R software packages: “corrplot”^67^, “FactoMineR”^68^, “missMDA”^69^ and “factoextra”^70^. Figures were made using SigmaPlot Version 11.0 (Systat Software, San Jose, CA).

## Electronic supplementary material

Supplementary information

## Data Availability

The datasets generated during and/or analysed during the current study are available from the corresponding author on reasonable request.
