# Supplementary material for: Aphid infestation in the phyllosphere affects primary metabolic profiles in the arbuscular mycorrhizal hyphosphere
Source: Sci Rep. 2018 Sep 27;8:14442. doi: 10.1038/s41598-018-32670-1 (PMC6160425; doi:10.1038/s41598-018-32670-1)
Supplement: Supplementary file 1 — Supplementary information [file 41598_2018_32670_MOESM1_ESM.pdf]

Supplementary information

Aphid infestation in the phyllosphere affects primary metabolic profiles in the arbuscular mycorrhizal hyphosphere

Carmina Cabral[1], Bernd Wollenweber[1], Carla António[2], Ana Margarida Rodrigues[2] and Sabine Ravnskov*[1]

[1] Aarhus University, Department of Agroecology, Forsøgsvej 1, DK-4200 Slagelse, Denmark

[2] Plant Metabolomics Laboratory, Instituto de Tecnologia Química e Biológica António Xavier-Universidade Nova de Lisboa (ITQB NOVA), Avenida da República 2780-157 Oeiras, Portugal

*Corresponding author

Email: sabine.ravnskov@agro.au.dk

Phone: +45 87 15 81 36

Fax: +45 87 15 60 82

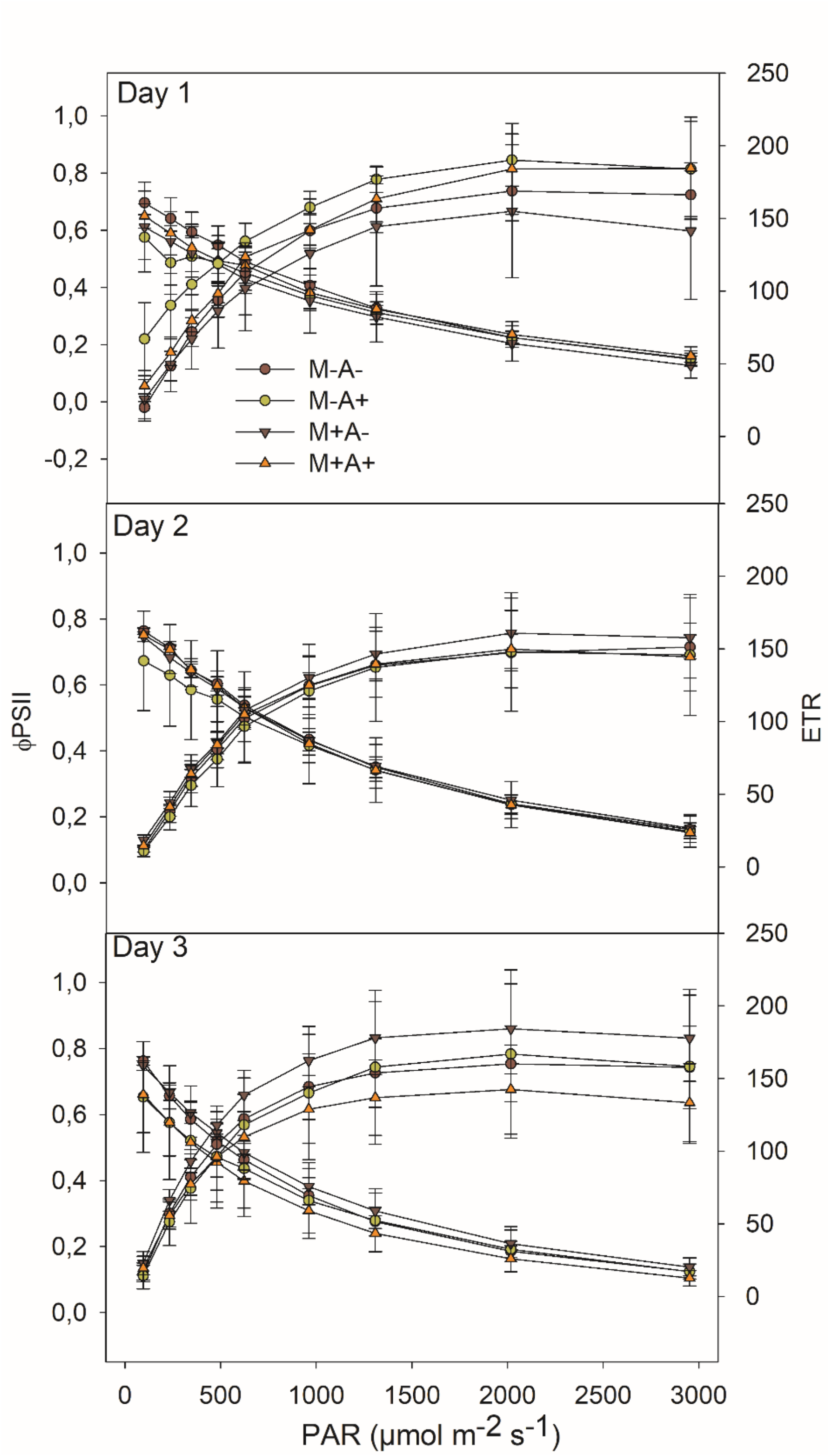

Supplementary Fig. S1 - Light-use efficiency parameters (ΦPSII: effective quantum yield; ETR: electron transfer rate) assessed in faba bean plants (*Vicia faba* L.). AM networks via the donor plant were (M+) established in receiver plants or not (M-). Donor plants in these systems were (A+) infested with aphids or not (A-). The parameters were measured during the aphid infestation period (Day 1 - 24, Day 2 - 48, Day 3 - 72H). Values are means (n=5).Error bars represent 95% confidence interval.

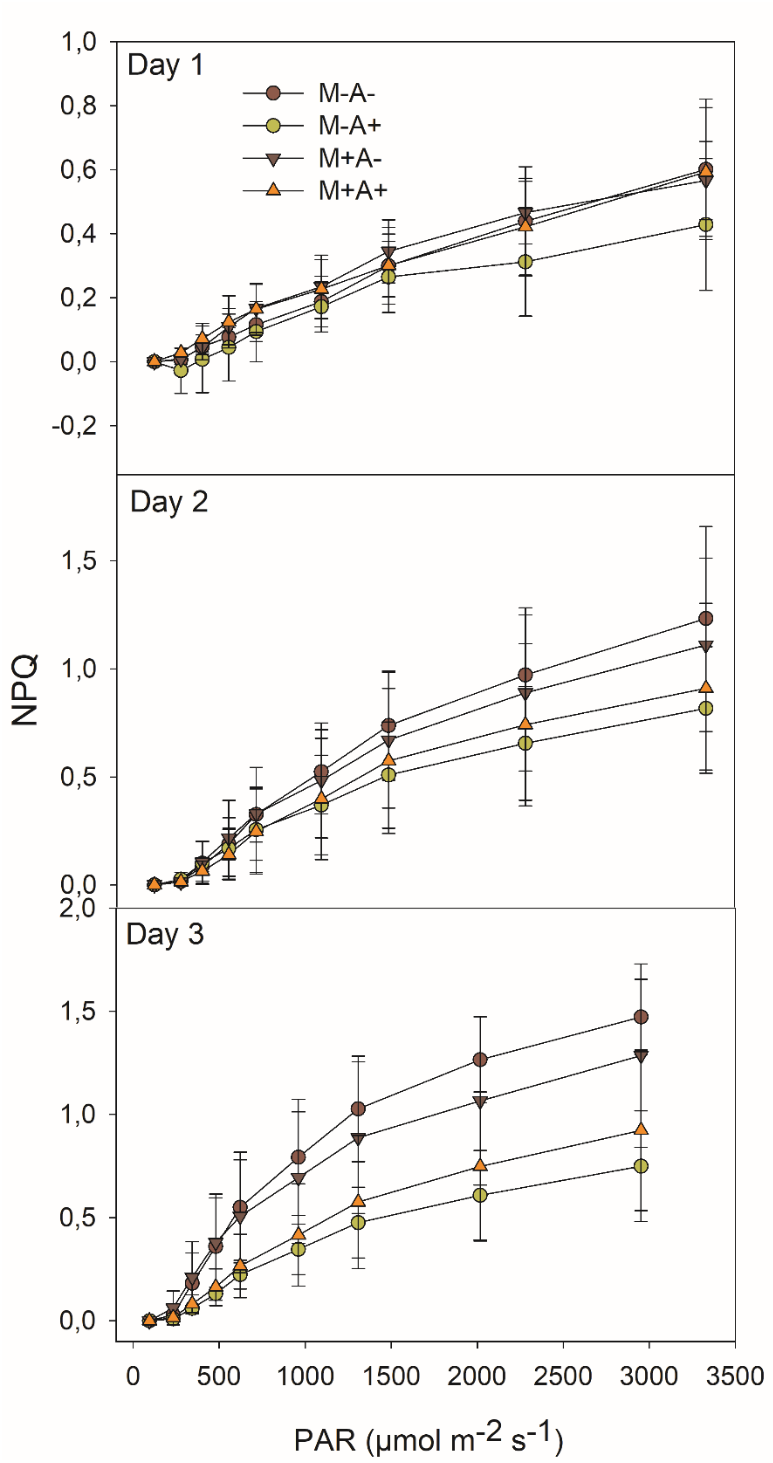

Supplementary Fig. S2 - Light-use efficiency parameters (NPQ: non-photochemical quenching) assessed in faba bean plants (Vicia faba). AM networks via the donor plant were (M+) established in receiver plants or not (M-). Donor plants in these systems were (A+) infested with aphids or not (A-). The parameters were measured during the aphid infestation period (Day 1 - 24, Day 2 - 48, Day 3 - 72H). Values are means (n=5).Error bars represent 95% confidence interval.

Supplementary Table S1 – Fold changes of primary metabolic profiles relative to M-A- treatment, normalised to the internal standard (ribitol) and dry weight of the samples, in undisturbed root-free compartments. AM networks via the donor plant were (M+) established in receiver plants or not (M-). Donor plants in these systems were (A+) infested with aphids or not (A-). EXT ID: external database ID from public reference library (CHEBI).Significant *P*-values (<0.05) are in bold. Values are means ± standard error (SE). n=5. [1]In each row, followed by the same letter are not significantly different by Tukey's HSD with a *P*-value fdr (false discovery rate) correction.

**Classes**	**Metabolites**	**EXT ID**	**M-A-**	**M- A+**	**M+ A-**	**M+ A+**	**ANOVA**		
							**AMF**	**Aphids**	**AMF*Aphids**
Amino acid	Serine	CHEBI:17799	1.00±0.25 a[1]	2.02 ± 0.98 a	2.01 ± 0.47 a	0.90 ± 0.28 a	0.81	0.64	0.33
Sugars	Fructose	CHEBI:48095	1.00±0.15 a	1.42 ± 0.30 a	0.62 ± 0.40 a	1.09 ± 0.10 a	0.39	0.30	0.95
	Fucose	CHEBI:18287	1.00±1.00 a	1.41 ± 0.08 a	1.10 ± 0.21 a	1.09 ± 0.185 a	0.66	0.36	0.29
	Galactose	CHEBI:28260	1.00±0.24 b	1.29 ± 0.14 b	1.02 ± 0.18 b	3.07 ± 0.25 a	**0.014**	**0.0005**	**0.0022**
	Glucose	CHEBI:17634	1.00±0.24 a	1.37 ± 0.10 a	1.02 ± 0.19 a	1.18 ± 0.55 a	0.72	0.35	0.72
	Rhamnose	CHEBI:26546	1.00±0.19 a	1.33 ± 0.12 a	1.06 ± 0.53 a	1.06 ± 0.18 a	0.64	0.43	0.44
	Sucrose	CHEBI:17992	1.00±0.45 b	0.78 ± 0.32 b	0.82 ± 0.55 b	3.76 ± 1.39 a	0.15	0.16	0.06
	Trehalose	CHEBI:16551	1.00±0.12 a	1.72 ± 0.30 a	1.06 ± 0.17 a	1.04 ± 0.28 a	0.24	0.19	0.16
Others	Phosphoric Acid	CHEBI:26078	1.00±0.15 b	2.09 ± 0.49 a	1.31 ± 0.34 ab	0.96 ± 0.28 b	0.35	0.28	0.06
	Urea	CHEBI:16199	1.00±0.15 a	1.30 ± 0.31 a	1.00 ± 0.14 a	0.89 ± 0.04 a	0.41	0.64	0.39

Supplementary Table S2 – Fold changes of primary metabolic profiles relative to M-A- treatment, normalised to the internal standard (ribitol) and dry weight of the samples, in disturbed root-free compartments. AM networks via the donor plant were (M+) established in receiver plants or not (M-). Donor plants in these systems were (A+) infested with aphids or not (A-). EXT ID: external database ID from public reference library (CHEBI). Significant *P*-values (<0.05) are in bold. n.d. – not determined. Values are means ± standard error (SE). n=5. [1]In each row, values followed by the same letter are not significantly different by Tukey's HSD with a *P*-value fdr (false discovery rate) correction.

**Classes**	**Metabolites**	**EXT ID**	**M-A-**	**M- A+**	**M+ A-**	**M+ A+**	**ANOVA**		
							**AMF**	**Aphids**	**AMF*Aphids**
Amino acid	Serine	CHEBI:17799	1.00±0.13 a[1]	n.d.	0.84±0.10 a	0.56±0.08 a	0.17	0.22	n.d
Sugars	Fructose	CHEBI:48095	1.00±0.13 a	0.96±0.15 a	0.69±0.69 a	0.66±0.04 a	0.18	0.89	0.98
	Fucose	CHEBI:18287	1.00±1.00 a	0.99±0.16 a	1.01±0.10 a	0.74±0.10 a	0.48	0.39	0.40
	Galactose	CHEBI:28260	1.00±0.12 a	0.93±0.18 a	0.76±0.06 a	0.79±0.15 a	0.23	0.89	0.72
	Glucose	CHEBI:17634	1.00±0.12 a	0.98±0.20 a	0.82±0.07 a	0.69±0.11 a	0.15	0.67	0.72
	Rhamnose	CHEBI:26546	1.00±0.16 a	0.89±0.14 a	1.03±0.11a	0.68±0.10 a	0.64	0.15	0.41
	Sucrose	CHEBI:17992	1.00±0.21 a	0.55±0.11 a	0.37±0.05a	0.59±0.25 a	0.13	0.64	0.14
	Trehalose	CHEBI:16551	1.00±0.11 a	0.77±0.12 a	1.09±0.17 a	0.73±0.10 a	0.75	0.06	0.63
Others	Fumaric Acid	CHEBI:18012	1.00±0.62 a	n.d.	1.43±0.62 a	0.37±0.08 a	0.94	0.05	n.d
	Phosphoric Acid	CHEBI:26078	1.00±0.21 ab	0.59±0.08 b	1.18±0.33 a	0.65±0.14 ab	0.50	**0.01**	0.86
	Urea	CHEBI:16199	1.00±0.14 a	0.43±0.16 a	1.16±0.27 a	0.51±0.04 a	0.58	**0.04**	0.76

**Metabolomics Standards Initiative Compliant Metadata**
According to:
Lisec, J., Schauer, N., Kopka, J., Willmitzer, L. & Fernie AR. (2006). Gas chromatography mass spectrometry-based metabolite profiling in plants. Nature Protocols, 1(1), 387–396. doi:10.1038/nprot.2006.59.
Fiehn, O. et al. (2007). Minimum reporting standards for plant biology context in metabolomics studies. Metabolomics 3(3), 195-201. doi:10.1007/s11306-007-0068-0.
Sumner, L. W. et al. (2007). Proposed minimum reporting standards for chemical analysis. Metabolomics 3(3), 211-221. doi:10.1007/s11306-007-0082-2.
"Fernie, A. R. et al. (2011). Recommendations for Reporting Metabolite Data. The Plant Cell 2(7), 2477-2482. doi 10.1105/ tpc.111.086272.<br>The Plant Cell July 2011 vol. 23 no. 7 2477-2482"

**BioSource**		
	Species	*Rhizophagus irregularis grown in 1:3 soil:sand mixture*
	Genotype	not applicable
	Organ	External mycelium
	Organ specification	not applicable
	Amount	4g per sample
**Growth**		
	Support	1:3 soil:sand mixture
	Location	Greenhouse
	Plot design	5 randomized blocks
	Light Period	16/8 h photoperiod (500 µmol m^{-2} s^{-1} light intensity)
	Humidity day	50%
	Humidity night	70%
	Temperature (day)	24ºC
	Temperature (night)	20ºC
	Watering	once daily
	Nutritional	no fertilization applied to growth media
	Date of plant establishment	20-01-2017
**Treatment**		
	Abiotic Treatment	not applicable
	Biotic Treatment	Aphis fabae
	Dose	30 adult aphids per plant
	Duration	84 hours
**Harvest**		
	Date	16th of March 2017
	Time	10:00 AM
	Growth Stage	9 week-old plants
	Metabolism quenching	quickly frozen in liquid nitrogen (i.e. shock freezing) and stored at -80 °C
	Harvest method	

	Storage	at - 80 °C until processed
**Sample Processing, Extraction, and Protocols**		
	Tissue processing	Hyphae in 1:3 soil:sand mixture was lyophilized, afterwards ground ion a ball-mill and homogenised.
	Replicate sampling and analyses	5 biological replicates
	Extraction of Samples: GC-MS	methanol: water: chloroform solution containing 0.2 mg mL^{-1} ribitol (IS) was used for extracton of polar metabolites for 40 min at 70 °C, 1000 µL of the polar fraction was evaporated
	Extract concentration: GC-MS	dried polar extracts were derivatized with 40 µL of 20 mg/mL of methoxyamine hydrochloride in pyridine followed by TMS derivatization using 70 µL of N-methyl-N-trimethylsilyltrifluoroacetamide and 20 µL of a mixture of fatty acid methyl esters (FAMES)
	Extract clean-up	none
	Extract storage	at - 80 °C
**GC-MS instrument**		
Gas Chromatography	Sample preparation	samples were used as described above
	Auto injector	MultiPurpose Sampler MPS, Gerstel, Germany
	Chromatography Instrument	6890N Agilent, Böblingen, Germany
	Separation column	VF-5MS column (Varian Inc., 30 m-lenght, 0.25 mm-inner diameter, and 0.25 µm-film thickness)
	Separation parameters	the injection temperature was 230 °C. The initial temperature of the oven (85 °C) increased at a rate of 15 °C min^{-1} up to a final temperature of 360 °C
Mass Spectrometry		
	Instrument	Pegasus III TOF, Leco Instruments, St. Joseph, USA
	Sample Introduction	GC-MS
	Ionization	electron ionization
	Polarity	positive ionization
	Mass Analyzer	time-of-flight
	Data acquisition	the transfer line to the mass spectrometer and ion source were set to 250 °C. After a solvent delay of 180 sec mass spectra were scanned from *m/z* 70-600 with the acquisition rate of 20 spectra s^{-1}
Instrument performance		

and Method Validation		
	Instrument calibration	internal mass calibration was performed using Agilent calibrant (PFTBA)
	Mass Resolution	unit mass
	Mass Accuracy	nominal mass
	Quantification	relative quantification by internal standard and sample dry weight (using fresh weight/dry weight ratio reported in Batista-Santos et al. 2015)
	Variation	based upon the internal standard
	Sample Replications	1 analytical (same analytical sample preparation) and 6 biological (same experimental condition) replicates
	Internal Standard	ribitol
	Quality Controls	FAMES internal standard markers and a QC control which consists of the same 1:3 soil:sand and hyphae material as each sample in every run.
	Blanks	multiple blanks and wash analyses were included in the experimental design
Data Preprocessing		
	File format	data were exported in a net.cdf format using Agilent MSD ChemStation
	Pre-processing details	data files were deconvoluted using an automated mass spectral deconvolution and identification system AMDIS (NIST, Gaithersburg, USA)
	Statistics	Data was normalized by dividing each peak intensity by the corresponding internal standard peak intensity, followed by dividing by the dry weight of each sample. Log10 transformation allowed the distributions to fit the assumptions of the ANOVA ($p<0.05$), followed by a Fisher's LSD post-hoc analysis. All statistical analsysis were done in R software.
Metabolite Identifications		
	unknown compounds	not applicable
	identified compounds	metabolites were identified by mass spectral matching against the published FAME MDN35 Library, from Golm Metabolome Database (http://gmd.mpimp-gol.mpg.de/download/) by using AMDIS and TagFinder 4.0 software
